# Salvage Stabilization via Transthoracic Approach for Congenital Vertebral Malformations in a Toy Breed Dog: A Case Report

**DOI:** 10.3390/ani16050719

**Published:** 2026-02-25

**Authors:** Hyeyeon Cheong, Jaegwan Cha

**Affiliations:** Oasis Veterinary Surgical Center, Suwon 16507, Republic of Korea; green@oasisvet.kr

**Keywords:** congenital vertebral malformation, toy breed, transthoracic approach, spinal stabilization, revision surgery, Pomeranian

## Abstract

This case report describes the successful management of a 7-month-old Pomeranian with congenital vertebral malformation (CVM) causing kyphosis and progressive myelopathy, characterized by non-ambulatory paraparesis, decreased postural reactions, and increased spinal reflexes in the hind limbs. Initial management involved a dorsal approach for decompression and stabilization; however, the patient failed to regain hindlimb function. Following two failed surgical attempts due to implant failure and progressive instability, a salvage stabilization was performed using a transthoracic approach. Stabilization was achieved using titanium screws and polymethyl methacrylate (PMMA) applied to the vertebral bodies. The patient exhibited marked postoperative improvement, regaining weight-bearing ability within one week and achieving a normal gait by four weeks. At the 19-month follow-up, the dog maintained normal ambulatory function with no recurrence of neurological deficits. While the transthoracic approach is well-documented in brachycephalic breeds, this is the first report describing its successful application in a toy breed. This case highlights the transthoracic approach as a highly effective salvage technique, offering a novel solution when conventional dorsal elements are irreversibly compromised due to the extremely diminutive and fragile nature of toy breed vertebrae.

## Simple Summary

Congenital vertebral malformations are spinal deformities that can cause paralysis in dogs. While surgery is often needed, it can be very difficult in tiny “toy” breeds like Pomeranians because their vertebrae are diminutive and fragile. This report describes a case of a 7-month-old Pomeranian that underwent two standard surgeries through the back (dorsal approach) to fix a spinal deformity, but both failed. We then performed a third “salvage” surgery, approaching through the chest (transthoracic approach) to stabilize the spine from the side. This method allowed us to use stronger bone for screw fixation. The dog recovered its walking ability completely. This case shows that the chest approach can be a successful alternative for saving toy breed dogs when standard back surgeries fail.

## 1. Introduction

Congenital vertebral malformations (CVMs) are developmental anomalies of the spine, frequently affecting the thoracic region. While commonly recognized in screw-tailed brachycephalic breeds such as French Bulldogs, English Bulldogs, Pugs, and Boston Terriers, these malformations also occur in toy breeds like Maltese, Pomeranians, and Yorkshire Terriers [1,2,3,4]. Such deformities—including kyphosis, lordosis, and scoliosis—often exacerbate during growth, potentially leading to vertebral canal stenosis and spinal instability. These structural changes can result in spinal cord injury caused by either static or dynamic compression. Most dogs affected by vertebral body malformations exhibit clinical symptoms, such as ataxia, paresis, and incontinence, within the first year of life [5].

A Cobb angle exceeding 35° has been shown to have the highest sensitivity and specificity for predicting clinical signs in affected dogs [3,6]. As these dogs continue to grow, neurological symptoms may worsen, necessitating surgical intervention. However, surgical management of CVMs is often fraught with complications and inconsistent outcomes, particularly in skeletally immature dogs with soft bone and abnormal anatomy [7,8]. These challenges are even more pronounced in toy breeds, where the small size of the vertebrae increases surgical complexity.

Two main surgical approaches have been described: the transthoracic approach for stabilization [9,10] and the dorsal approach, which often requires more extensive dissection [1,2,7,11]. Historically, the dorsal approach has been preferred due to difficulties in accessing the thoracic region [4,9,10]. This case report describes an improved stabilization method using a transthoracic approach as a revision procedure after the failure of a dorsal approach in a toy breed dog, outlining the surgical technique and the successful clinical outcome.

## 2. Case Description

### 2.1. Clinical Presentation and Initial Findings

**Figure 1:**
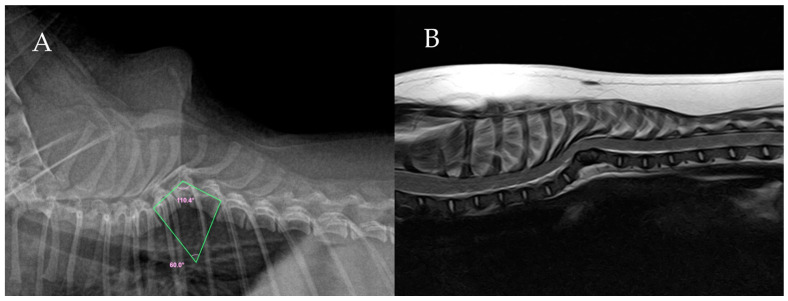
Preoperative imaging of the thoracic spine. (**A**) Lateral radiographic view showing severe kyphosis centered at T7 with a Cobb angle of 60.0°. (**B**) Sagittal T2-weighted magnetic resonance image showing spinal cord compression at the level of the malformation.

A 7-month-old intact female Pomeranian weighing 2.4 kg presented with a 2-month history of ataxia. Initially, the dog dragged its hind legs and frequently fell. Upon initial presentation, the dog exhibited non-ambulatory paraparesis but was able to maintain a standing posture when supported. Neurological examination revealed absent conscious proprioception and exaggerated spinal reflexes in the hindlimbs, consistent with an upper motor neuron (UMN) lesion localized to the T3-L3 spinal cord segments. Superficial and deep pain perception remained intact in both pelvic limbs. Cranial nerve examination and mentation were unremarkable, and spinal hyperesthesia was not distinctly evident. Despite unknown oral medications prescribed by a local clinic, the symptoms gradually worsened. Complete blood count (CBC), serum chemistry, electrolyte analysis, and coagulation tests were within normal limits. Radiographs confirmed T7 vertebral hypoplasia, leading to thoracic vertebral malformation and misalignment. Kyphosis was observed, and the Cobb angle was calculated at 60° (Figure 1). All Cobb angles throughout the clinical course were precisely measured to a precision of 0.1 degrees using dedicated digital veterinary templating software (vPOP PRO version 3.3.2, VetSOS Education Ltd., Shrewsbury, UK).

### 2.2. Imaging and 3D Printing

Upon admission, Magnetic Resonance Imaging (MRI) and Computed Tomography (CT) scans were performed. General anesthesia was induced with butorphanol (0.2 mg/kg IV) and midazolam (0.1 mg/kg IV), followed by propofol (4 mg/kg IV) induction and maintenance with sevoflurane and oxygen. Sagittal, transverse, and dorsal MR images were obtained using a 1.5 Tesla MRI system (Vantage Elan, Canon Medical Systems, Otawara, Japan), with sequences including T2-weighted, T2 STIR, T1-weighted, and T1 contrast-enhanced images. The MRI revealed mild intervertebral disk (IVD) degeneration at T6–T7 and T7–T8, with reduced dorsoventral vertebral canal diameter at the T7 level. The most severe spinal cord compression was observed at T6–T7.

Cone-beam CT scans (PHION, Nano Focus Ray, Jeonju, Republic of Korea) were performed concurrently, and the images were used for preoperative planning using three-dimensional (3D) printing technology. DICOM files from the CT scan were imported into Osirix MD (Pixmeo, Geneva, Switzerland) and converted to STL files in Autodesk Meshmixer (Autodesk, San Rafael, CA, USA), which were then printed using an SLA 3D printer (Form 2, Formlabs, Somerville, MA, USA). The 1:1 scale anatomical 3D-printed model was utilized for preoperative simulation and visual reference to evaluate vertebral dimensions, visualize optimal implant trajectories, and select appropriate implant sizes. Additionally, the physical model allowed the surgical team to visually confirm the extremely narrow pedicles and limited bone stock prior to surgery.

### 2.3. First Surgical Procedure and Outcome

Surgery was conducted using a previously described decompression and vertebral stabilization technique [1,7]. Anesthesia was administered following the imaging protocol. Cefazolin (22 mg/kg IV) was given every 90 min as prophylactic antibiotics, and remifentanil (0.1 mg/kg/min CRI) was used for intraoperative analgesia. A dorsal midline incision was made over the thoracic vertebrae, and a dorsal laminectomy was performed from T6 to T8 using nitrogen-powered burrs. A subcutaneous fat graft was placed over the defect to prevent adhesions. Six positive-thread pins (IMEX Veterinary Inc., Longview, TX, USA) were inserted bilaterally into T6, T7, and T8.

**Figure 2:**
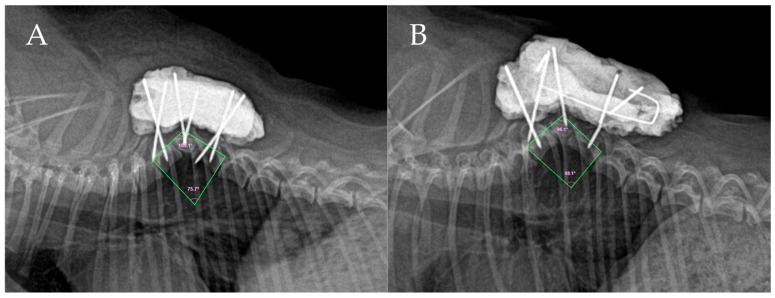
Postoperative lateral radiographs following the initial and revision dorsal stabilization surgeries. (**A**) Lateral radiograph obtained after the first surgical procedure, showing dorsal stabilization using threaded pins and PMMA. The Cobb angle is measured at 75.7°, indicating an increase in kyphosis. (**B**) Radiograph taken 18 days after the first revision surgery, demonstrating severe progressive kyphotic collapse with the angle worsening to 88.1° and subsequent implant breakage.

Due to the extremely diminutive and narrow pedicles characteristic of a toy breed, achieving bicortical purchase was technically challenging. Following pin insertion, stabilized with polymethyl methacrylate (PMMA) bone cement (Spine-Fix, Teknimed SAS, Vic en Bigorre, France) was applied over the exposed portions of the pins to complete the bilateral fixation construct (Figure 2A). The precise trajectories and depths of the pins were evaluated using postoperative CT imaging obtained immediately after the surgery. The retrospective analysis revealed the following placement: at T6, the right pin engaged only the pedicle, while the left pin penetrated the pedicle and approximately 50% of the vertebral body depth. At the malformed T7, the right pin was anchored in the transverse process, and the left pin engaged the pedicle and vertebral body (approx. 70% depth), but with a slight breach into the vertebral canal due to the narrow safety corridor. At T8, the right pin achieved bicortical purchase, but the left pin breached the medial pedicle wall, encroaching into the canal. The severe kyphotic angulation at T7 created a steep cranial slope, making visualization and optimal pin trajectory at T6 particularly difficult. Postoperatively, the dog was prescribed firocoxib (8 mg/kg SID PO), pregabalin (3.5 mg/kg BID PO), and amoxicillin-clavulanate (13.5 mg/kg BID PO). Physical therapy, including transcutaneous electrical nerve stimulation (TENS) around the surgical site, was also administered. However, because the pins could not be placed with sufficient depth or bicortical engagement, the PMMA-pin construct could not withstand the biomechanical forces, ultimately leading to construct failure. Consequently, the dog did not recover hindlimb function, and non-weight-bearing hind limb status persisted for 17 days postoperatively.

### 2.4. First Revision Surgery and Outcome

Eighteen days after the initial surgery, a second surgical procedure was performed because the patient showed no neurological improvement in hindlimb ataxia, and a postoperative CT scan obtained immediately after the first surgery suggested that the left threaded pins at T7 and T8 had breached the medial pedicle wall. This finding raised significant concerns regarding iatrogenic spinal cord injury and potential implant loosening, which were considered the primary causes of the persistent neurological deficits. During the revision, the PMMA was partially removed using a pneumatic burr. Intraoperative inspection confirmed that the left threaded pins at T7 and T8 were loosened due to insufficient bone purchase. Therefore, only the left pins at T7 and T8 were extracted to prevent further neurological damage and address the implant failure. The bilateral pins at T6 and the right pins at T7 and T8 appeared secure and were retained. However, due to the minimal bone stock remaining in the small vertebrae, there was no safe corridor to insert new pins on the left side of T7 or T8 without risking iatrogenic fracture. As an alternative salvage attempt to indirectly enhance stability without placing new pins into the compromised bone, a Kirschner wire (K-wire) was passed through the T9 spinous process and bent bilaterally, and a short transverse K-wire was placed cranially between the existing bilateral PMMA masses. Additional PMMA was applied over these wires to create a connected, box-like dorsal augmentation construct (Figure 2B).

On the second postoperative day, discharge was noted at the surgical site. Empirical antibiotic therapy with amikacin (15 mg/kg SC) and clindamycin (13 mg/kg BID PO) was immediately initiated to manage a suspected surgical site infection. Bacterial culture and susceptibility testing were declined by the owner due to financial constraints. Neuro-muscular electrical stimulation (NMES) was also employed to prevent muscle atrophy in the hind limbs. Despite these interventions, this augmented dorsal construct failed to provide adequate biomechanical support against the progressive kyphotic forces. Eighteen days post-revision, follow-up radiographs revealed implant breakage and a severe progression of the deformity, with the Cobb angle worsening significantly to 88.1°. Consequently, the hind limbs remained non-weight-bearing, necessitating a fundamentally different surgical approach.

### 2.5. Second Revision Surgery (Transthoracic Approach)

Due to the lack of neurological improvement and the worsening Cobb angle, indicating progressive vertebral instability, a third surgery was necessary to enhance stabilization. This surgery took place 31 days after the second procedure, utilizing a transthoracic approach. The dog was placed under general anesthesia and positioned in right lateral recumbency, with the thoracic dorsal side elevated by approximately 30° using a bean bag positioner for improved visualization of the surgical field.

The procedure began with a dorsal approach to remove the previously implanted hardware. The surgical site was reopened, and the PMMA used in the previous surgery was carefully removed with a pneumatic burr. Each fixation pin was then removed, except for a broken pin at T8, which remained in place due to its short length. After completing a thorough lavage of the area, the dorsal site was closed using standard surgical techniques. The next step involved a thoracotomy performed through the seventh left intercostal space. The thoracic cavity was accessed using two Weitlaner retractors to expand the intercostal space for adequate visualization. The left lung and aorta were protected using moist gauze packs during the procedure. Paravertebral soft tissues were carefully dissected and retracted to expose the vertebral bodies.

**Figure 3:**
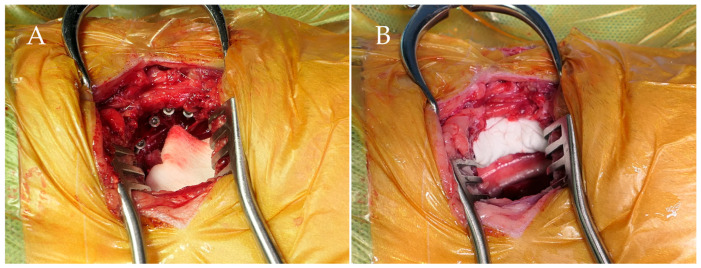
Intraoperative photographs of the transthoracic stabilization. (**A**) Titanium self-tapping screws inserted into the thoracic vertebral bodies. (**B**) Application of polymethyl methacrylate (PMMA) over the screw heads to achieve rigid fixation.

Drilling was initiated at T5, the most cranial vertebral body unaffected by the malformation at T7. A 23-gauge hypodermic needle was used to mark the drilling site, and a 1.0 mm drill guide was positioned perpendicularly to the vertebral body. Drilling was carried out with a 1.0 mm drill, stopping once penetration through the opposite cortex was detected. Titanium self-tapping screws, 1.2 mm in diameter, were inserted into the vertebral bodies, leaving 5–6 mm of the screw heads and shafts exposed (Figure 3A). Once the screws were secured, PMMA was applied around the exposed screw heads to ensure solid fixation (Figure 3B). The area was irrigated with sterile saline throughout the process to control temperature and prevent heat-related damage as the PMMA cured. A thoracic drain tube was placed in the thoracic cavity, and the surgical site was closed using standard procedures to complete the operation.

### 2.6. Final Outcome and Long-Term Follow-Up

**Figure 4:**
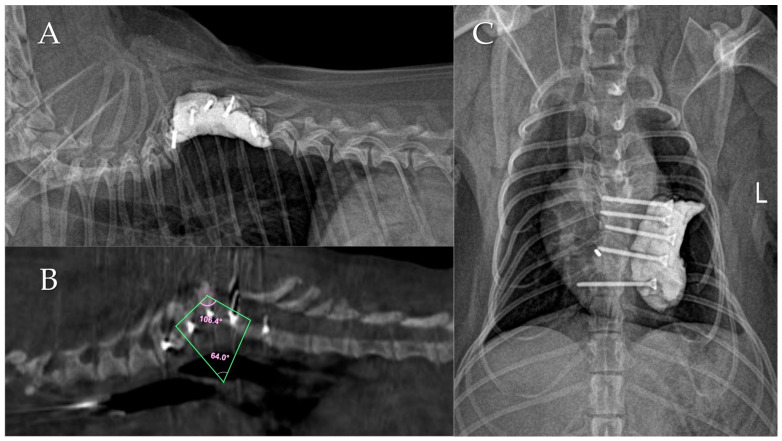
Postoperative imaging following the final transthoracic salvage stabilization. (**A**) Lateral radiograph and (**B**) sagittal cone-beam computed tomography (CBCT) image demonstrating the stabilized segment. Due to the extensive PMMA mass obscuring the vertebral endplates on standard radiographs, CBCT provided the definitive Cobb angle measurement of 64.0°. (**C**) Ventrodorsal radiograph showing the stable final construct with bilateral screws and PMMA. L indicates the left side.

Immediately following the second revision surgery, the severely worsened Cobb angle of 88.1° (caused by the previous implant failure) was reduced to 64.0° (Figure 4). Only minimal thoracic effusion was observed through the thoracic drain, which led to the removal of the drain tube on the third postoperative day. Neurological improvements in the hind limbs began shortly after surgery. By the seventh postoperative day, the dog had regained the ability to bear weight on its hind limbs. The neurological deficits improved rapidly, and by four weeks post-surgery, the dog exhibited a normal gait. At the 19-month follow-up after the final surgery, the dog maintained normal walking ability without any recurrence of neurological symptoms. Notably, no further changes in the Cobb angle were observed during this period.

## 3. Discussion

Congenital vertebral malformations (CVMs) in dogs frequently occur in the thoracic region, leading to secondary neurological dysfunction due to spinal canal stenosis and instability [7,12,13]. The severity and progression of symptoms typically dictate whether conservative or surgical treatment is warranted [12]. While conservative management has been recommended for young dogs with non-progressive symptoms, recent studies have shown that conservative treatment often results in poor outcomes, especially in progressive cases [14]. Consequently, surgical intervention becomes necessary for cases of severe or progressive myelopathy [7].

In humans, kyphoscoliosis leads to dynamic spinal cord compression due to spinal instability [15,16], and in dogs, dynamic compression has also been suggested as a primary mechanism of spinal cord injury in CVMs [9]. Vertebral malformations may result from disruption of the ossification centers during development, leading to angular deformities such as kyphosis, which cause spinal canal stenosis, spinal cord compression, and instability. It is thought that repeated dynamic spinal cord damage, caused by instability, compression, or a combination of both, leads to secondary neurological dysfunction [5,13].

Many studies have shown that spinal canal stenosis is not a significant factor in the pathophysiology of kyphosis-related cases, and positive outcomes have been achieved with stabilization alone, without additional decompression [2,9,10]. This suggests that stabilization might be sufficient for treating CVMs in certain cases, although breed-specific considerations are necessary. Most of the studies on CVMs focus on brachycephalic breeds, such as French Bulldogs, English Bulldogs, and Pugs. In these breeds, the prevalence of CVMs is surprisingly high, reaching up to 75% to 100% in French Bulldogs [12,13,17,18]. In English Bulldogs, the prevalence ranges from 73.2% to 97.3%, while in Pugs, one or more thoracic hemivertebrae are found in 17.6% of cases [4,18]. This suggests a genetic basis, especially in screw-tailed breeds like French Bulldogs, where the condition is associated with a mutation in the DISHEVELLED 2 gene [19]. Interestingly, Pugs, which do not carry this genetic mutation, present a different type of vertebral malformation, ventral hypoplasia, commonly linked to kyphosis [18]. These findings indicate that the pathophysiology of thoracic hemivertebra may differ between Pugs and screw-tailed brachycephalic breeds [4].

Despite the high prevalence of CVMs in screw-tailed brachycephalic breeds, they are rarely reported in toy breed dogs. The pathophysiology and surgical treatments in toy breeds may differ, and there is little data on effective treatments for CVMs in these dogs. This underscores the need for individualized surgical approaches based on breed and case-specific factors.

Several surgical techniques for CVMs have been developed, focusing on decompression, stabilization, or a combination of both [2,8]. In our case, the initial surgical method involved vertebral stabilization using pins and PMMA via a dorsal approach, as described in previous studies [1,7]. While these studies generally reported favorable outcomes, complications such as implant failure, pin breakage, and loosening have been noted. Similarly, Charalambous et al. observed pin encroachment and implant loosening in dogs treated with Steinmann pins [2]. Consistent with these reports, our patient experienced implant loosening and pin breakage after the first and second surgeries, leading to persistent instability.

Consequently, revision surgery via the previous dorsal approach was deemed unfeasible. In addition to the inherent anatomical challenges posed by the malformed thoracic vertebrae—specifically the downward slope of the cranial aspect—the dorsal elements were now severely compromised. The first revision surgery revealed that the dorsal bone stock was already exhausted, which indicated that any further attempts to re-engage the pedicles would have posed an unacceptable risk of iatrogenic fracture. Thus, the transthoracic approach was not merely an alternative but an anatomical necessity for salvage. It provided access to the pristine ventral and lateral aspects of the vertebral bodies, allowing for the engagement of screws into untouched, higher-quality bone stock.

However, accurately drilling and inserting screws at the correct angle into the thoracic vertebrae of toy breed dogs presents significant challenges [1,20]. Although this was not an issue in our case, improper techniques can lead to significant complications, such as iatrogenic spinal cord injury or damage to nerve roots and vertebral arteries, which may result in serious medical consequences [21,22].

While advanced 3D-printed patient-specific surgical guides could theoretically optimize implant placement during dorsal stabilization [11,23,24], we utilized a simple 3D-printed anatomical model for visual planning in this case. Elford et al. demonstrated 96.7% accuracy in pedicle screw placement across six brachycephalic dogs, although two screws breached the spinal canal in a severely kyphotic case [11]. Similarly, Violini et al. found that 84% of screws were optimally placed, but 15% penetrated the pedicle wall in 20 dogs [24]. Ultimately, regardless of the planning method, the diminutive and narrow pedicles of toy breeds present a hard physical limit to achieving robust bone purchase dorsally. Both studies used dorsal approaches, and no research exists yet on using 3D-printed guides for transthoracic stabilization. Although 3D-printed guides may be beneficial for this approach, further studies are needed. Given the small margin for error in toy breeds, the surgeon’s skill remains critical [20].

There have been two prior studies reporting vertebral stabilization using intercostal thoracotomy. Mathiesen et al. demonstrated favorable outcomes in six Pugs using bilateral intercostal thoracotomy and String of Pearls (SOP) plate fixation, without any decompression procedures or vertebral realignment [9]. In contrast, Mariné et al. utilized a unilateral intercostal thoracotomy in 10 dogs, including Pugs and French Bulldogs, using monocortical screws and PMMA for stabilization [10]. A key difference in Mariné’s study was the goal of partial correction of the deformity angle, which was not part of the approach in either Mathiesen’s cases or our case.

In Mariné’s approach, traction screws and a Caspar distractor were employed to realign the vertebrae before PMMA fixation. Despite not performing spinal decompression, they reported favorable outcomes [10]. However, in our case, spinal cord decompression had already been accomplished via dorsal laminectomy during the initial surgery. Consequently, the potential benefit of anatomical realignment on relieving spinal cord compression was considered minimal. Furthermore, applying mechanical distraction forces to the extremely diminutive and fragile vertebral bodies of a toy breed introduces a severe risk of iatrogenic bone fracture or implant pull-out. Therefore, we prioritized rigid in situ stabilization over risky anatomical realignment. The lateral intercostal incision (thoracotomy) provided sufficient surgical visibility without significant hindrance during the procedure.

Following the revision surgery and successful stabilization, the clinical symptoms improved rapidly, underscoring the importance of stabilization in the treatment of CVMs. Regarding deformity correction, while the final postoperative Cobb angle (64.0°) did not fully return to the absolute initial presentation angle (60.0°), the transthoracic stabilization successfully corrected the severe kyphotic progression (88.1°) that resulted from the dorsal construct failure. It is important to note that this improvement from 88.1° to 64.0° was not achieved through forceful mechanical distraction. Rather, the 88.1° angle represented a dynamic, unsupported collapse of the destabilized segment. Positioning the patient in lateral recumbency under general anesthesia eliminated gravitational loading, allowing for spontaneous postural reduction in the spine to a neutral resting position, which was then rigidly secured in situ.

This case report represents the first documented successful application of the transthoracic stabilization technique in a toy breed dog, particularly as a salvage procedure for failed dorsal stabilization. As discussed, the dorsal approach in these diminutive patients is often severely hindered by the microscopic scale of the pedicles and a steep cranial slope of the malformed vertebrae, predisposing standard constructs to catastrophic mechanical failure [1,2,20]. By demonstrating that the transthoracic approach provides access to pristine, high-quality ventral bone stock when dorsal elements are compromised, this report offers a critical and novel solution for complex revision surgeries in this underserved patient population. While the pathophysiology of CVMs in toy breeds remains to be fully elucidated, our findings strongly suggest that this alternative approach can successfully overcome the limitations of conventional dorsal implants and provide rigid, long-lasting stability.

## 4. Conclusions

In conclusion, our case demonstrates that in toy breed dogs, a transthoracic approach for stabilization after failed dorsal surgeries can provide good long-term outcomes and should be considered as an alternative to dorsal approaches, particularly when anatomical challenges make traditional methods difficult. This approach may offer a more stable and long-lasting solution for CVM management in toy breed dogs.

## Data Availability

The data presented in this study are available on request from the corresponding author.

## Abbreviations

The following abbreviations are used in this manuscript:

CVM	Congenital vertebral malformation
PMMA	Polymethyl methacrylate
MRI	Magnetic resonance imaging
CT	Computed tomography
3D	Three-dimensional
IVD	Intervertebral disk
UMN	Upper motor neuron
CBC	Complete blood count
CRI	Constant rate infusion
TENS	Transcutaneous electrical nerve stimulation
NMES	Neuromuscular electrical stimulation
SOP	String of Pearls
